# Characterization and QTL Mapping of a Major Field Resistance Locus for Bacterial Blight in Rice

**DOI:** 10.3390/plants11111404

**Published:** 2022-05-25

**Authors:** Jae-Ryoung Park, Chang-Min Lee, Hyeonso Ji, Man-Kee Baek, Jeonghwan Seo, O-Young Jeong, Hyun-Su Park

**Affiliations:** 1 Crop Breeding Division, National Institute of Crop Science, Rural Development Administration, Wanju 55365, Korea; icd0192@korea.kr (J.-R.P.); cropas@korea.kr (C.-M.L.); baekmg@korea.kr (M.-K.B.); rightseo@korea.kr (J.S.); joyoung@korea.kr (O.-Y.J.); 2 Department of Agricultural Biotechnology, National Institute of Agricultural Sciences, Rural Development Administration (RDA), Jeonju 54874, Korea; jhs77@korea.kr

**Keywords:** bacterial blight disease, rice, breeding, QTL, SNP

## Abstract

Bacterial blight (BB) disease, caused by *Xanthomonas oryzae* pv. *oryzae* (*Xoo*), is among the major factors that can cause rice yields to decrease. To address BB disease, researchers have been looking for ways to change pesticides and cultivation methods, but developing resistant cultivars is the most effective method. However, the resistance and genetic factors of cultivars may be destroyed due to the emergence of new *Xoo* species caused by recent and rapid climate changes. Therefore, breeders need to identify resistance genes that can be sustained during unpredictable climate changes and utilized for breeding. Here, *qBBR11*, a quantitative trait locus (QTL) for resistance to BB disease, was detected in KJ (Korea *Japonica* varieties) 11_067 to KJ11_068 on chromosome 11 in a population derived by crossing JJ (Jeonju) 623 and HR(High resistant)27,195, which possess similar genetic backgrounds but different degrees of resistance to BB disease. *qBBR11* was reduced from 18.49–18.69 Mbp of chromosome 11 to 200 kbp segment franked. In this region, 16 candidate genes were detected, and we identified 24 moderate-impact variations and four high-impact variations. In particular, high-impact variations were detected in *Os11g0517800* which encode the domain region of *GCN2* which is the eIF-2-alpha kinase associated with the resistance of abiotic/biotic stress in rice. In JJ623, which is moderately resistant to BB disease, a stop codon was created due to single nucleotide polymorphism (SNP). Therefore, compared with HR27195, JJ623 has weaker resistance to BB disease, though the two have similar genetic backgrounds. The results suggest that variation in the *qBBR11* region regulates an important role in improving resistance to BB diseases, and *qBBR11* is useful in providing an important resource for marker-assisted selection to improve mechanisms of resistance to BB disease.

## 1. Introduction

Rice is used as a staple food by half of the world’s population and is responsible for most of the calories consumed [1,2]. The world’s population has rapidly increased in recent years, so solving the food shortage problem and ending hunger have been the most prioritized breeding goals of breeders for a long time [3]. However, due to recent and rapid climate change and unpredictable environments, species differentiation is accelerating, and preparing for an unpredictable future is difficult with the existing resistant cultivars [4,5].

Bacterial blight (BB) disease, which is caused by *Xanthomonas oryzae* pv. *oryzae* (*Xoo*), is a major cause of severe losses in rice yields, mainly in Asia, and is among the most devastating diseases [6]. In particular, Asia is a region in which more than 90% of the world’s rice production is cultivated and exported, so the development of BB disease-resistant varieties and the discovery of resistance genes is an essential goal [7,8]. BB diseases interfere with the growth of rice through the proliferation of bacteria, which invade through the hands and wounds of individuals. Bacteria multiply in the xylem and phloem, interfering with the movement of water and nutrients, causing the leaves to turn white, and photosynthesis is hindered, which causes a loss in yields and a deterioration in grain quality [9].

BB diseases in rice cause various types of damage throughout the rice growth period, and the degree of damage varies [10]. As the growth of rice progresses, the incidence of BB disease increases; the incidence is highest in the heading stage, and the degree of damage is severe [11]. In addition, because BB is also greatly influenced by the environment, more damage by BB disease occurs in tropical regions than in temperate regions, and yield losses of 2–74% occur in tropical regions depending on the cultivation period, environmental conditions, growth period and kind of cultivars [12]. Therefore, rice has established complex molecular mechanisms that respond to *Xoo* [13].

Various methods have been used to try to control BB diseases. The most widely used method is to apply chemical control [14]; the application of chemical pesticides is the main cause of environmental pollution, and it creates an irreversibly damaged habitat by simultaneously causing soil and water pollution [15]. Additionally, because the differentiation of the *Xoo* species that causes BB diseases is very rapid, using chemical pesticides to control the disease is ultimately very impractical [16]. Therefore, breeding resistant cultivars using BB disease resistance genes is considered the most economical and environmentally friendly method and has become the main breeding strategy [17].

Currently, there are more than 40 genes for resistance to BB disease that have been studied in rice [18,19]. These resistance genes are concentrated on chromosome 4 and chromosome 11, and among these genes, eight resistance genes located on chromosome 4, *Xa1*, *Xa2* [20], *Xa12*, *Xa14* [21], *Xa25*, *Xa20(t)*, *Xa31(t)* [22], and *Xa38* [23], are distributed. Twelve resistance genes, such as *Xa3*/*Xa26*, *Xa4* [24], *Xa10*, *Xa21*, *Xa22*, *Xa23* [25], *Xa30(t)*, *Xa32(t)* [26], *Xa35(t)*, *Xa36(t)*, *Xa39*, and *Xa40* [27], are located on chromosome 11, and these genes have been successfully cloned using a map-based cloning strategy. Among these resistance genes, *Xa3* is expressed from the seedling stage, then the resistance intensity becomes stronger as growth progresses, and the resistance to bacteria is widespread and resilient [28]. In addition, *Xa21* has broad resistance to *Xoo*, and its expression becomes stronger after the heading date [29,30]. In addition, *Xa3*, *Xa4*, *Xa7*, *Xa21*, and *Xa23* have been used mainly to develop BB disease-resistant rice cultivars because of their broader spectrum of resistance [31,32,33].

Although many studies related to the development of resistant varieties by a single gene have been conducted, the resistance conferred by a single resistance gene can be effective for a specific *Xoo* bacterium, but due to the continuous evolution of the bacteria, the resistance by a single gene is easily disrupted [34]. However, in contrast, quantitative gene resistance of quantitative trait locus (QTL) is not bacteria specific, and QTLs are considered to have a wide range of resistance [35]. A breeding system that develops new cultivars through the identification of new resistance genes and complementation with known resistance genes will be applied as an important method for breeding sustainable resistant cultivars by improving resistance.

In this study, we report QTL mapping, which is important in inducing resistance to BB diseases. To evaluate BB disease resistance, various BB disease isolates were used, and by applying these results, quantitative loci related to BB disease resistance were identified through QTL mapping using Kompetitive allele-specific PCR (KASP) marker sets. In addition to the known genes that are related to BB disease resistance, allele types were analyzed through candidate gene screening and sequencing to identify new major BB disease resistance genes, and the possible effect on resistance in rice was confirmed.

## 2. Results

### 2.1. Evaluation of BB Resistance Using the RIL Population

JJ623 is derived through marker-assisted backcrossing that is based on an interspecific cross and has a model background. Since the resistance does not become 100% identical through crossbreeding, it is not possible to obtain the optimal state. Sindongjin has only *Xa3* and is resistant to K1, K2, and K3 but is sensitive to K3a. In addition, HR27195 has both *Xa3* and *Xa21* and is highly resistant to K1, K2, K3, and K3a. However, despite having both *Xa3* and *Xa21*, JJ623 has strong resistance to K1, K2, and K3 but moderate resistance to K3a. Therefore, we backcrossed HR27195 to improve the resistance of BB and analyzed the QTLs involved in resistance to all *Xoo* bacteria. JJ623 and HR27195 are rice varieties and K1, K2, K3 are pathotypes of *Xoo*. The *Xa3* and *Xa21* genotypes were analyzed for 90 JJ623/HR27195 F_2_ individuals, and advanced development was performed by single seed descent.

### 2.2. Development of the Mapping Population and R-Gene Inheritance

**Figure 1:**
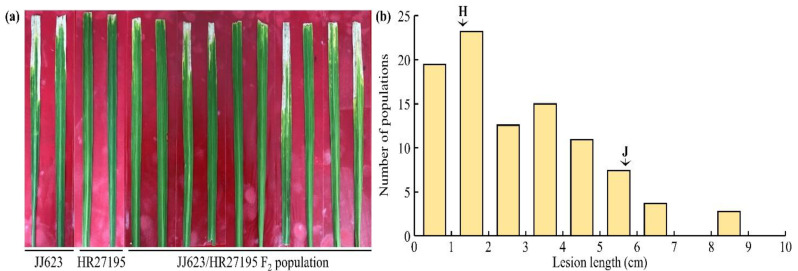
Frequency distribution and symptoms of lesion length induced by *Xanthomonas oryzae* pv. *oryzae* (*Xoo*) in the F_2_ line derived from JJ623/HR27195 crossing, JJ623, and HR27195. (**a**) Disease phenotypes after *Xoo* inoculation in JJ623, HR27195, and JJ623/HR27195 F_2_ lines by *Xoo*. JJ623 is moderately resistant to Xoo infection, but HR27195 is strongly resistant to Xoo. However, various phenotypes from susceptible to resistant were investigated in the JJ623/HR27195 F_2_ line. (**b**) The lesion length of JJ623 shows a moderate resistance of 5.6 cm and that of HR27195 a strong resistance of 1.3 cm. When *Xoo* was infected on the JJ623, HR27195 and JJ623/HR27195 F_2_ lines, various lesion lengths were investigated for each line. For the normal distribution, each of the resistance phenotype, moderate resistance phenotype, and susceptibility phenotype showed a left-biased normal distribution. H: HR27195, J: JJ623.

To determine the genes related to HR27195 BB resistance, a total of 90 F_2_ lines were generated from a cross between HR27195, an *R*-gene donor, and JJ623, which is moderately resistant to BB. At 14 days after *Xoo* inoculation in the HR27195, JJ623, and F_2_ lines, lesion length was measured for each line (Table 1 and Figure 1a). HR27195 and JJ623 had the same allele combination of the BB disease resistance gene *Xa3* + *Xa21* (Supplementary Figure S1). However, 24 days after inoculation, the average lesion length of JJ623 was 3.8 cm, indicating moderate resistance, whereas the average lesion length of HR27195 was 1.3 cm, indicating strong resistance. The mean lesion length of the F_2_ lines was 2.8 cm, and the lesion length had a wide range of 0.5–8.7 cm. Additionally, the distribution of the lesion length in the F_2_ lines was not normal (Figure 1b), but at the same stage, the major agricultural traits resulted in a normal distribution pattern (data not shown). When each line was divided into resistance and sensitivity using the lesion length, the line was divided into 29 resistant lines and 61 sensitive lines (Table 2), which were suitable for the expected phenotypic separation ratio of 1:3 (*X*^2^ = 2.50, and *P* = 0.113). Through lesion length evaluation of F_2_ lines, *Xoo* resistance confirmed that a single resistance gene was involved.

### 2.3. Analysis of QTLs for BB Resistance

**Figure 2:**
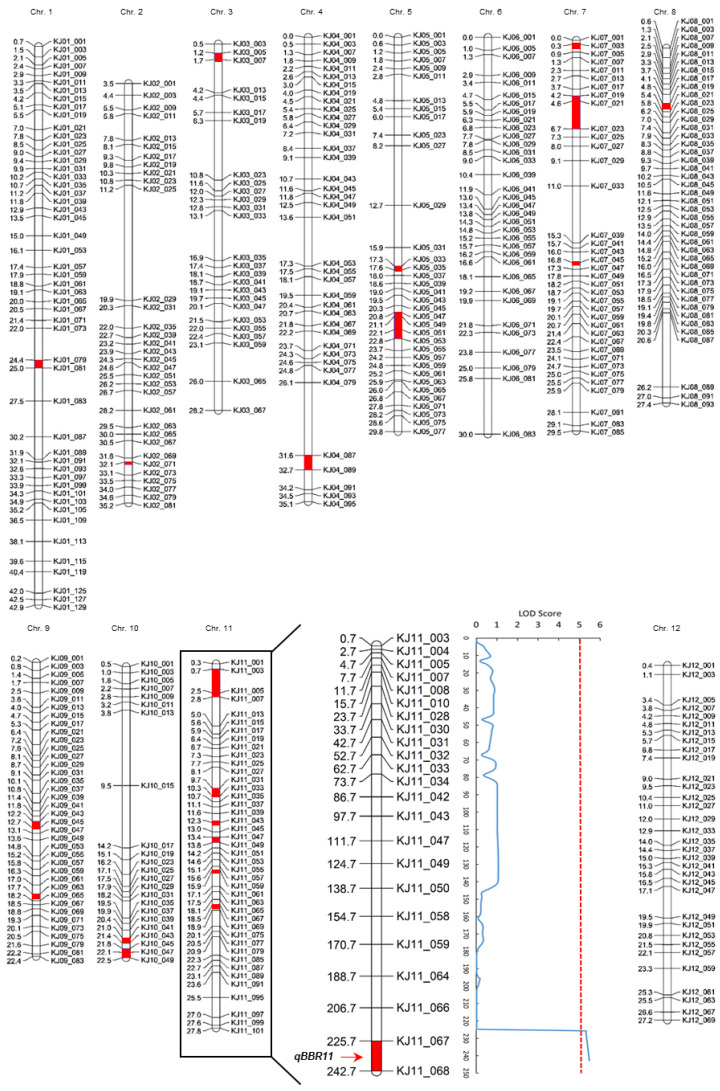
Genetic linkage map constructed based on the KASP marker. (**a**) Distribution of polymorphic KASP markers of JJ623 and HR27195 on 12 rice chromosomes. Among the 888 KASP markers, polymorphisms of JJ623 and HR27195 were analyzed in the remaining chromosomes except for chromosome 12, and these regions are marked in red. (**b**) LOD contour of the QTL mapped region using lesion length by *Xoo*. An LOD score of 5.47 was detected at 17.0 cM of KJ11_067 to KJ11_068, showing the region of the corresponding chromosome. The KASP marker name is shown on the right side of the chromosome, and the number on the left shows the genetic distance of the markers in centimorgans (cM).

**Figure 3:**
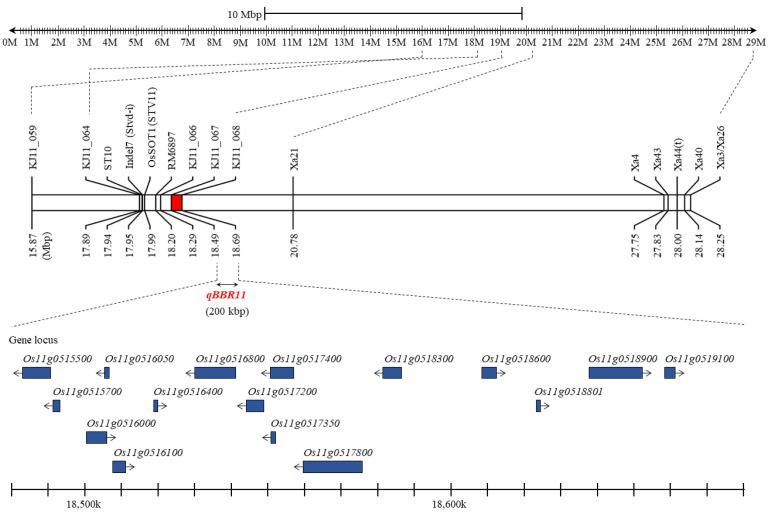
Annotation of candidate genes related to resistance to bacterial blight (BB) by physical mapping. QTLs related to BB resistance were detected in the 200 kbp region between KJ11_067 and KJ11_068 of chromosome 11. In *qBBR11* detected on chromosome 11, there are 16 candidate genes annotated by comparison with the sequence of Nipponbare. In the region adjacent to *qBBR11*, the gene *OsSOT1* related to rice stripe virus resistance and ST10, Indel7, and RM6897, molecular markers that detect rice stripe virus resistance, were identified together, and the BB resistance gene *Xa21* was also identified.

BB disease resistance-related QTLs were mapped to linkage maps that were constructed using KASP marker sets (Figure 2). Of the 888 KASP markers, only 77 KASP markers were polymorphisms for JJ623 and HR27195, and among these, the precision of one marker (KJ10_045) in the F_2_ line was low, so a total of 76 KASP markers were used to analyze the QTLs involved in BB disease resistance. Seventy-six KASP markers were distributed on 12 chromosomes in rice. QTL mapping was performed using lesion length data, and when inclusive composite interval mapping (ICIM) and an empirical threshold of LOD >3.0 were applied, *qBRR11* (BB resistance QTL on chromosome 11) was detected on chromosome 11 (Table 3). *qBRR11* was detected in KJ11_067 to KJ11_068 of chromosome 11, and the LOD score was 5.47. This position corresponds to 18.49–18.69 Mbp of chromosome 11, which can account for 24.5% of the lesion length variation in the population and was derived from HR27195. The QTL region associated with blight resistance was narrowed into 200 kbp segments of chromosome 11 (Figure 3). When the sequence of Nipponbare was used as a reference for the target region, 16 candidate genes were identified (Table 4). Additionally, in the near region of *qBBR11*, the rice stripe virus resistance gene *OsSOT1* (17.90 Mbp) was searched, and markers for detecting rice stripe virus resistance ST10 (17.94 Mbp), Indel7 (17.95 Mbp), and RM6897 (18.20 Mbp) were adjacent. The BB resistance gene *Xa21* (20.78 Mbp) is also located in the region.

### 2.4. Analysis of the Genetic Segregation Ratio of the Resistance Gene

The combined effect of *qBBR11,* as detected by QTLs, that was related to BB disease resistance was analyzed. When haplotype analysis was performed, the following haplotypes were distinguished: *qBBR11^JJ623^*, *qBBR11^JJ623^qBBR11^HR27195^*, and *qBBR11^HR27195^*. In the F2 lines, the separation ratio of each haplotype was 24:40:26 (Supplementary Figure S2), which is appropriate for the expected phenotypic ratio of 1:2:1 (*X*^2^ = 1.20, and *p* = 0.549). The lesion lengths of the three haplotypes, *qBBR11^JJ623^*, *qBBR11^JJ623^qBBR11^HR27195^*, and *qBBR11^HR27195^*, were 5.6 ± 1.14 cm, 2.8 ± 1.92 cm, and 1.3 ± 0.27 cm, respectively. The *qBBR11^JJ623^* allele type increased lesion length and ultimately increased susceptibility to BB diseases.

### 2.5. Candidate Genes and Sequencing Analysis

**Figure 4:**
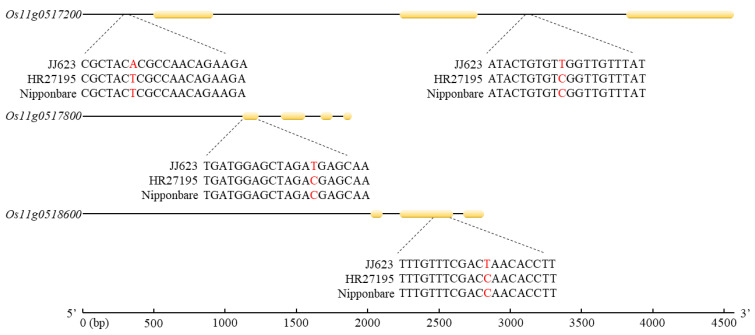
Sequence difference analysis of *Os11g0517200*, *Os11g0517800*, and *Os11g0518600* of JJ623 and HR27195 by SNP. Among the candidate genes related to BB resistance detected by *qBRR11*, SNPs were identified from *Os11g0517200*, *Os11g0517800*, and *Os11g0518600*. In *Os11g0517200*. SNPs were detected in two regions, but they were all identified in the intron region; however, SNPs were detected in exon in *Os11g0517200* and *Os11g0517800*.In particular, due to the SNP detected in *Os11g0518600*, JJ623, which is moderately resistant to Xoo, forms a stop codon and cannot translate eIF-2-alpha kinase GCN2, which is important for Xoo resistance.

When *qBBR11* detected by QTL mapping using lesion length was analyzed by designating the Nipponbare sequence as a reference, there were 16 candidate genes in the *qBBR11* genomic region. Of the 16 candidate genes, 3 were non-protein coding transcripts, and 7 were conserved hypothetical proteins; one similar to transport inhibitor response 1 protein, one similar to serine palmitoyltransferase, one ribosomal protein L31 domain-containing protein, one similar to maturase K protein, and similar to OSIGBa0121O24.2 protein, and one with a domain region of GCN2, which is an eIF-2-alpha kinase, were encoded. To identify the sequence variation in candidate genes, the sequences of 16 candidate genes were compared in two parents, JJ623 and HR27195. A total of 1081 single nucleotide polymorphisms (SNPs) and insertions and deletions (InDels) were discovered in *qBBR11*, among which 24 variations with moderate impact and 4 variations with high impact were found. All 24 moderate impact variations were missense variants. Four high-impact variations were distributed in three candidate genes as follows: two in *Os11g0517200*, one in *Os11g00517800*, and one in *Os11g0518600*. In *Os11g0517200*, SNPs were detected as T→A at 18,544,678 bp and T→A at 18,548,438 bp of JJ623 when compared with Nipponbare. All of these SNPs were detected by base substitution in the intron region. (Figure 4). *Os11g00517800* was a base substitution in the exon region, and an SNP was detected from 18,561,020 bp of JJ623 to G→A. CGA, which is the reference sequence, encodes arginine, but in JJ623, a stop codon is created as CGA→TGA is substituted due to SNP. In addition, *Os11g0518600* was a base substitution in an exon region, and SNPs were detected as C→T at 18,610,615 bp of JJ623. CAA, which is the reference sequence, codes for glutamine, but in JJ623, a stop codon is created by replacing CAA→TAA due to SNP.

## 3. Discussion

BB disease is one of the factors that has directly led to the decrease in rice yield that has been widely observed around the world [36]. The cultivation of BB disease-resistant cultivars is an important factor for eliminating hunger [37]. To breed actual BB disease-resistant cultivars, the most popular method is to map genes that are related to BB disease resistance and apply them to breeding [38]. However, due to recent rapid climate change and unpredictable environments, pathogenic variation continues to appear in *Xoo*, which breaks down the resistance of the currently cultivated rice [39]. Therefore, rather than breeding a cultivar with resistance by a single gene, it is necessary to diversify the germplasms of BB disease-resistant varieties by breeding a cultivar with two or more effective resistance genes [40,41].

In this study, the BB disease-resistant cultivar HR27195 was selected as a resistance gene donor, and an F_2_ line was developed through cross with JJ623, a moderate-resistance cultivar. QTLs involved in BB resistance were detected using this population. After crossing Sindongjin and HR27195, JJ623 was bred by backcrossing Sindongjin twice to recover the genetic background of Sindongjin. Sindongjin was recognized as an excellent cultivar due to its high yield and improved grain quality [42], but the BB R-gene possesses only *Xa3* [43]. *Xa3* is resistant to the *Xoo* bacteria K1, K2, and K3 but is sensitive to K3a. In 2003, a new *Xoo* bacteria, K3a, was isolated in Korea, and a reduction in yield became a very serious problem due to K3a [33]. Therefore, to introduce the K3a resistance gene *Xa21* into Sindongjin, HR27195 was selected as the R gene donor, and HR27195 was crossed with Sindongjin. As a result, JJ623, including both *Xa3* and *Xa21*, was introduced, and it was strongly resistant to K1, K2, and K3. However, JJ623 was moderately resistant to K3a.

Here, QTL mapping was performed to analyze the cause of the moderate resistance to K3a that occurred despite the accumulation of *Xa3* and *Xa21*. The F_2_ line used for QTL mapping consisted of 90 populations. Individuals from the 90 F_2_ line of JJ623/HR27195 used for QTL mapping were matched with a single gene with a dominant gene action in which the resistance gene was isolated in a ratio of 1:3. The HR27195 used in this study showed resistance to all tested bacteria used when bioassayed using 4 BB isolates of different bacteria in Korea. However, while HB27195 showed broad resistance (lesion length 1.3 ± 0.27 cm), JJ623 showed resistance to K1, K2, and K3 but was moderately resistant to K3a, and JJ623 had the same *Xa3* + *Xa21* genotype as HB27195. Despite this, the lesion length for K3a was 3.8 ± 1.14 cm, indicating moderate resistance. Despite having the *Xa3* + *Xa21* genotype combination, which is currently known to be strongly involved in BB disease resistance, QTL mapping and sequence analysis were performed to analyze the cause of the moderate resistance of JJ623. In this study, the population number constrained for QTL linkage research or association mapping related to BB disease resistance was relatively small. However, it has been reported that even if the size of the mapping group is small, the target region can be detected sufficiently, and considerable time and money can be saved [44]. In addition, QTL regions that have already been detected using a small population number are effectively widely used as breeding materials. In this study, a genetic map was constructed using the KASP marker. The KASP marker is a codominant allele that can identify SNP [45] and InDel variation when analyzing the mating separation ratio for marker assisted selection (MAS) and is applied to single-step genotyping technology [46]. SNPs are abundant in the plant genome and have become a powerful tool for genome selection [47]. The current SNP is expected to largely replace the simple sequence repeat (SSR) marker and most molecular markers that have been used in the preparation of high-density genetic maps for rice, wheat, and barley in the past [48,49,50]. Often, when mapping QTLs to a specific region to improve agronomic characteristics, a genetic map has been constructed using cultivars that were derived through crossings between species with a distant genetic distance [51]. Therefore, the currently designed and developed SSR markers and restriction fragment length polymorphism (RFLP) markers for QTL mapping of rice detect polymorphisms between *Indica* and *Japonica*, which have very long genetic distances [52,53]. Therefore, these molecular markers have many limitations in detecting polymorphisms in cultivars with high genetic similarity [54]. Thus, in this study, KASP markers that can be used for genetic analysis of *Japonica* rice cultivars with high genetic similarity were applied [55]. For JJ623 and HR27195, which have similar genetic backgrounds, polymorphisms were analyzed in 76 KASP markers out of 888 KASP markers. Despite the similar genetic backgrounds of JJ623 and HR27195, a high QTL was detected on chromosome 11 with an LOD score of 5.47, and the size of this region was 200 kbp, which was very narrow and down to the size of a region similar to fine mapping [56,57]. In addition, candidate genes were selected, and sequencing analysis was performed to improve the accuracy of the detected region and determine genetic differences. Based on QTL analysis, *qBBR11*, which is thought to be involved in BB disease resistance, was identified at the end of chromosome 11 in rice, and resistance increased even when *qBBR11^HR27195^* was heterozygous, but the homozygous type had the shortest lesion length, and resistance increased the most. qBBR11, which was determined to be a major resistance gene, has an LOD score of 5.47, and 24.5% of the phenotypic variation can be explained. BB disease resistance genes have been reported in various populations of rice cultivars through previous studies [58], and 26 dominant genes and 14 recessive genes were studied [7]. Among the 12 rice chromosomes, the genes are distributed on 10 chromosomes except for chromosomes 9 and 10, and genes for BB resistance are distributed intensively on chromosomes 4 and 11 [59].

In this study, *qBBR11*, a novel BB disease resistance-related QTL that has not been reported before, was newly mapped, new resistance-related candidate genes were screened on chromosome 11, and polymorphisms were analyzed through sequencing. The reason that BB disease-related resistance QTLs were detected at a different location from those of previous studies is because the group used in the study and the environmental factors when conducting the study are different, and the location is a result of the interaction between the phenotype and the genotype [60]. However, all relevant QTLs were detected in adjacent regions. *qBBR11* (1849–1869 Mbp) is located adjacent to *Xa21* (20.78 Mbp), *Xa4* (27.75 Mbp), *Xa43* (27.83 Mbp), *xa44(t)* (28.00 Mbp), *Xa40* (28.14 Mbp), *Xa3/Xa26* (28.25 Mbp) on chromosome 11, which has already been identified as a key factor in BB resistance. When analyzing the effect of *qBBR11* on BB disease resistance in the population developed for QTL mapping, the degree of resistance was established with the most certainty when *qBBR11^HR27195^* was homozygous. However, the level of *qBBR11^JJ623^* sensitivity was not serious, and the lesion length was 5.6 ± 1.14 cm, which was considered to indicate moderate resistance. To determine the effect of *qBBR11*, a candidate gene was detected, and sequencing analysis was performed. In *qBBR11*, 16 candidate genes related to BB disease resistance were screened, and this region was sequenced. Of the 28 found variations by SNP and InDel, 24 were moderately impacted variations, and 4 were highly impacted variations. Among the highly impacted variations, *Os11g0517200* is an intron, and *Os11g0518600* has a stop codon instead of arginine and glutamine due to SNP. *Os11g0518600* has a domain region of eIF-2-alpha kinase GCN2. GCN2 is a kinase that reduces protein biosynthesis by phosphorylating transcription initiators and regulates the abiotic/biotic stress response of plants [61,62]. In particular, in *Nicotiana tabacum*, phosphorylation of NteIF2A increases not only the expression of GCN2 but also the transcript level of plant hormones such as salicylic acid, azelaic acid, and methyl jasmonate, which are involved in various plant defense systems that are related to abiotic/biotic stress resistance [63]. Despite possessing *Xa3* and *Xa21*, which are currently reported to be most strongly involved in BB disease resistance, the different degrees of resistance between HB27195 and JJ623 suggest that sequence variation in *qBBR11* plays an important role. The results of this study indicate that a wide range of resistance exists among various *Xoo* strains that are involved in BB disease; in addition, the study provides useful information for understanding the molecular mechanisms involved in BB disease resistance and improving resistance.

## 4. Materials and Methods

### 4.1. Plant Material and Field Design

The mapping population consists of 90 F_2_ lines that were obtained through a cross of JJ623 (BB moderately resistant parent) and HR27195 (BB resistant parent). JJ623 breeding through the cross of Sindongjin (*Oryza sativa* L. spp. *Japonica* cv. Sindongjin) [43], which has a strong resistance to *Xoo* bacteria K1, K2, and K3 due to the *Xa3 R*-gene, and HR27195, which has strong resistance to K1, K2, K3, and K3a due to the *Xa3 + Xa21 R*-gene [43]. The JJ623/HR27195 F_2_ line was used to screen BB resistance QTLs and candidate genes through genotyping and phenotyping. JJ623, HR27195, JJ623/HR27195 F_2_ lines were cultivated and evaluated in the fields (36°6′41.54″ N, 128°38′26.17″ E) of the National Institute of Crop Sciences (NICS), Rural Development Administration (RDA), Wanju, South Korea, in 2019 and 2020. Plant materials were used for research in compliance with international guidelines and legislation provided by RDA in Korea and cultivated according to normal local practices. Before sowing, the seeds were soaked in darkness for 3 days at 33 °C using a seed disinfectant solution (Spotak, 25% Prochloraz, HANKOOKSAMGONG, Seoul, South Korea). Sowing was performed in the NICS field on 2 May 2019, and 30 April 2020, and transplanted into the field 30 days after sowing. The planting distance was 30 × 15 cm, and the fertilization amount was N-P_2_O_5_-K_2_O = 90-45-57 kg/ha, which was applied according to the standards of the Agricultural Science and Technology Research Survey Standard of RDA (rural development administration).

### 4.2. Bacterial Strains and Plant Inoculations

BB bacteria, including K1 (HB1013), K2 (HB1014), K3 (HB1015), and K3a (HB1009), were used to evaluate the resistance and susceptibility to BB. Each *Xoo* strain was cultured in PSA medium (10 g peptone, 10 g sucrose, 1 g glutamic acid, 16 g bacto agar, pH 7.0) at 28 °C in the dark for 3 days [64]. The cultured *Xoo* strains were diluted to an OD_600_ of 0.2 in distilled water using a spectrophotometer. The prepared *Xoo* strains were inoculated on each line using the leaf-clipping method [65], and the fully opened leaves were cut at the maximum tillering stage after the scissors were completely immersed in the *Xoo* strain suspension. Each line was evaluated after measuring the lesion length according to the standard evaluation methods of RDA Korea after 14 days of inoculation to evaluate the spectrum of the resistant group and the susceptible group [66]. When the lesion length was less than 3 cm, it was assigned to the resistant group; when the lesion length was 3–5 cm, it was assigned to the moderate group; and when the lesion length was larger than 5 cm, it was assigned to the susceptible group. For each line, leaf damage and lesion length by *Xoo* were measured in 5 leaves.

### 4.3. DNA Extraction and PCR Analysis

BioSprint 96 DNA Plant Kit (INDICAL BIOSCIENCE, Cat. SP947057, Leipzing, Germany) was used to extract genomic DNA from plant materials. Samples were ground using Tissue LyserII (QIAGEN, Cat. 85300, Hilden, Germany), and genomic DNA was extracted according to the manual provided in the BioSprint 96 DNA Plant Kit (INDICAL BIOSCIENCE, Cat. SP947057, Leipzing, Germany). The extracted DNA was evaluated for nucleic acid quantification and quality using a Nanodrop ND 1000 spectrophotometer (ThermoFisher, Cat. ND 1000, Waltham, MA, USA). PCR analysis was performed in a My-Genie 96 Thermal block (BIONEER, Cat. A-2040-3, Daejeon, Korea) using 10 ng of DNA template and AccuPower PCR PreMix (BIONEER, Cat. K-2018, Daejeon, Korea). The PCR profile was subjected to initial denaturation at 94 °C for 5 min, denaturation for 30 s at 94 °C, annealing at 55–60 °C for 30 s, extension at 72 °C for 30 s, and final extension at 72 °C for 5 min. For the PCR profile, the denaturation–annealing–extension process was performed for 35 cycles. The PCR product was confirmed using a UV transilluminator (BIORAD, Cat. 170–8070, Hercules, CA, USA) after electrophoresis on a 0.8% agarose gel containing EtBr (SIGMA, Cat. E1510, Saint Louis, MO, USA).

### 4.4. Genotyping and Linkage Mapping

To analyze the QTLs that are related to BB disease resistance, single nucleotide polymorphisms (SNPs) were detected using 888 KASP (Kompetitive Allele Specific PCR) markers. Of the 888 KASP markers, only 76 KASP markers had polymorphisms in their parents JJ623 and HR27195. For the KASP marker that was ultimately selected, linkage mapping was constructed using the linkage mapping software QTL IciMapping v4.1 [67]. The SNPs that were selected for linkage mapping were identified using the MAP functionality of lciMapping software. The marker distance of the linkage map was calculated by the recombination frequency by the Kosambi function, and LOD and input options were applied for grouping and anchoring of SNPs. To detect the QTLs that were related to BB disease resistance, the average lesion length of five leaves was used. The LOD score was used as a threshold for declaring the importance of QTLs, and QTLs were defined at *p* < 0.05.

### 4.5. Analysis of Putative Candidate Genes

The genomic sequence of the marker interval detected by QTL mapping was analyzed with FGENESH software (https://www.sofberry.com (accessed on 11 September 2021)) with reference to the genome sequence of Nipponbare (*Oryza sativa* L. spp. *Japonica* cv. Nipponbare). Open reading frames (ORFs) in the QTL region were searched based on the annotation databases of NCBI (https://www.ncbi.nlm.nih.gov/ (accessed on 15 September 2021)) and Gramene (https://www.gramene.org/ (accessed on 16 September 2021)). In addition, the putative functions of genes in the region of interest were annotated using the BLAST-P function of NCBI. In addition, the putative function of candidate genes present in the detected QTL region was described using RiceXpro (https://ricexpro.dna.affrc.go.jp/ (accessed on 18 September 2021)).

### 4.6. Haplotype Sequence Analysis

To search for single nucleotide polymorphisms (SNPs), the Illumina HiSeq2000 platform was used according to the method suggested by Kumagai, et al. [68], and Nipponbare’s sequence was used as a reference genome. Trimmomatic removal was performed for low-quality bases and adapter sequences from each read. The variants of each sample were called using GATK Haplotype Caller, the variants of each cultivar were combined with GATK Combine GVCFs, and finally, the genotype of each cultivar was designated with GATK Genotype GVCFs. Additionally, the GATK Variant Filtration and GATK Select Variants were set to ‘D < 5.0, FS > 50.0, SOR > 3.0, MQ < 50.0, MQ Rank Sum < −2.5, Read Pos Rank Sum < −1.0, Read Pos Rank Sum > 3.5’ to analyze the hard filtering of variants. Filtered mutations were annotated with rice genome annotation information using the RAP database (RAP-DB, https://rapdb.dna.affrc.go.jp/ (accessed on 18 September 2021)) [69]. For the finally selected variants, nucleotide diversity (π) and allele number frequency of alleles were calculated using vcftools (v0.1.13), and the polymorphism information content value was calculated through the frequency of alleles [70]. To analyze InDels in the QTL mapping region, a primer was designed using CLC Genomics Workbench (v6.0.1). In addition, the agronomically important gene list of RAP-DB was used to search for sequence variations in major agronomical genes.

## 5. Conclusions

In this study, *qBBR11*, a novel QTL region related to BB resistance, was identified by applying the KASP marker set. Several ORFs in *qBBR11* are closely related as candidate genes that can become *R*-genes involved in BB resistance. Sixteen candidate genes predicted to be involved in BB resistant in *qBBR11* were screened, and as a result of sequence analysis, SNPs were detected in *Os11g0517200*, *Os11g0517800*, and *Os11g0518600*. Among them, *Os11g0517800* encodes *eIF*-2-alpha kinase *GCN2* and is involved in BB resistance. However, when a stop codon is created due to SNP, *GCN2* is not translated, which has a negative effect on BB resistance. JJ623 and HR27195 both have the *Xa3* + *Xa21* combination allele, which regulates strong resistance against BB, but only the SNP generated in *qBBR11* determines whether it will become strongly resistant or moderately resistant. Therefore, if the *qBBR11* region is effectively utilized in the breeding program, it will be possible to cultivate a wide range of resistant varieties against *Xoo* and BB.

## Figures and Tables

**Table 1:** Phenotypic evaluation in leaves of Sindongjin, JJ623 and HR27195 inoculated with four pathotypes of *Xanthomonas oryzae* pv. *oryzae* (*Xoo*) induced bacterial blight (BB).

Cultivar	K1	K2	K3	K3a
Sindongjin	R	R	R	S
JJ623	R	R	R	MR
HR27195	R	R	R	R

JJ623 and HR27195 are rice varieties and K1, K2, K3 are pathotypes of *Xoo.* R, resistance; MR, moderately resistant; S, susceptible.

**Table 2:** Genetic analysis of the resistance response using lesion length for pathotype of *Xoo* K3a, a bacterium that induces BB of the F_2_ line derived between JJ623 and HR27195.

**Cross**	**Number of Resistance**	**Number of Susceptible**	**Total Number**	**Segregation Ratio**	***X* ^2^**	***p*-Value**
JJ623/HR27195	29	61	90	1:3	2.50	0.113

**Table 3:** Details of QTLs related to BB resistance from the F_2_ line derived between JJ623 and HR27195.

QTL	Chr.	Position (cM)	Physical Position (Mbp)	Left Marker	Right Marker	LOD ^z^	PVE ^y^(%)	Add ^x^	Dom ^w^
*qBBR11*	11	242.7	18.49–18.69	KJ11_067	KJ11_068	5.47	24.5	1.26	−0.09

^z^ Logarithm of the odds. ^y^ Phenotypic variation explained, a ratio that can explain the variation in lesion length in the JJ623/HR27195 F_2_ line. ^x^ Additive effect, (lesion length of *qBBR11^JJ623^*-lesion length of *qBBR11^HR27195^*)/2. ^w^ Dominant effect, Effect of the *qBBR11^JJ623^* allele dominantly on the *qBBR11^HR27195^* allele on lesion length, positive values of the additive effect indicate that alleles from HR27195 are in the direction of increases in the traits.

**Table 4:** Candidate genes associated with BB at *qBBR11* and variation-induced single nucleotide polymorphisms (SNPs) between JJ623 and HR27195.

Gene ID	Physical Position (bp)	Putative Function
*Os11g0515500*	18,485,308–18,490,843	Similar to transport inhibitor response 1 protein
*Os11g0515700*	18,493,370–18,494,393	Nonprotein coding transcript
*Os11g0516000*	18,503,539–18,507,701	Similar to Serine palmitoyltransferase (Fragment)
*Os11g0516050*	18,506,918–18,507,468	Nonprotein coding transcript
*Os11g0516100*	18,509,086–18,512,628	Ribosomal protein L31 domain containing protein.
*Os11g0516400*	18,520,372–18,521,065	Hypothetical conserved gene
*Os11g0516800*	18,531,653–18,541,501	Hypothetical protein
*Os11g0517200*	18,544,614–18,548,898	Hypothetical conserved gene
*Os11g0517350*	18,550,900–18,551,191	Similar to Maturase K (Fragment)
*Os11g0517400*	18,552,567–18,556,300	Conserved hypothetical protein
*Os11g0517800*	18,560,178–18,575,490	eIF-2-alpha kinase GCN2
*Os11g0518300*	18,581,847–18,585,958	Hypothetical protein
*Os11g0518600*	18,608,152–18,611,496	Conserved hypothetical protein
*Os11g0518801*	18,622,623–18,622,684	Nonprotein coding transcript
*Os11g0518900*	18,636,476–18,650,782	Conserved hypothetical protein
*Os11g0519100*	18,655,353–18,658,368	Similar to OSIGBa0132O24.2 protein

## Data Availability

The data presented in this study are available on request from the corresponding author.

## Supplementary Materials

The following supporting information can be downloaded at: https://www.mdpi.com/article/10.3390/plants11111404/s1, Figure S1: Genotyping analysis related to bacterial blight (BB) resistance to strains K1, K2, K3, and K3a causing BB in JJ623 and HR27195. (a) JJ623 is resistant to each strain of K1, K2, and K3, but only moderately resistant to K3a. However, HR27195 is resistant to all strains evaluated. (b) In JJ623 and HR27195, the lesion length for BB-inducing K3a was determined differently, but BB resistance-related genes *Xa3* and *Xa21* were amplified in both JJ623 and HR27195. M; DNA ladder, J; JJ623, H; HR27195. Figure S2: Frequency of *qBBR11* allele type in JJ623/HR27195 F_2_ line. In the F_2_ line, *qBBR11^JJ623^qBBR11^JJ623^*, *qBBR11^JJ623^qBBR11^HR27195^* (HE), and *qBBR11^HR27195^qBBR11^HR27195^* were separated 24:40:26, respectively. *qBBR11* in the F_2_ line was suitable for the theoretical separation ratio of 1:2:1 (*X*^2^ = 1.20, *P*-value = 0.549). The lesion lengths of *qBBR11^JJ623^qBBR11^JJ623^*, *qBBR11^JJ623^qBBR11^HR27195^*, *qBBR11^HR27195^qBBR11^HR27195^* were 4.1 cm, 2.8 cm, and 1.6 cm, respectively. The allele type of *qBBR11^JJ623^* regulate as an increase in lesion length.
