# Supplementary material for: Characterization and QTL Mapping of a Major Field Resistance Locus for Bacterial Blight in Rice
Source: Plants (Basel). 2022 May 25;11(11):1404. doi: 10.3390/plants11111404 (PMC9182613; doi:10.3390/plants11111404)
Supplement: Supplementary file 1 [file plants-11-01404-s001.zip › plants-1709553-supplementary.pdf]

Supplementary Materials

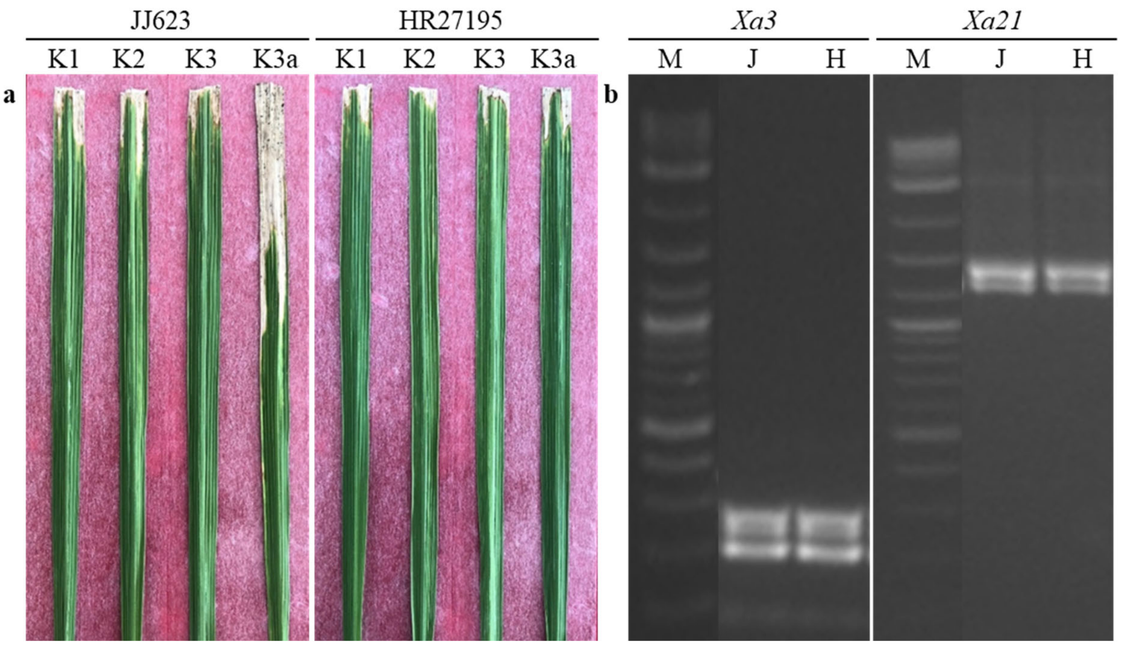

Figure S1. Genotyping analysis related to bacterial blight (BB) resistance to strains K1, K2, K3, and K3a causing BB in JJ623 and HR27195. **a** JJ623 is resistant to each strain of K1, K2, and K3, but only moderately resistant to K3a. However, HR27195 is resistant to all strains evaluated. **b** In JJ623 and HR27195, the lesion length for BB-inducing K3a was determined differently, but BB resistance-related genes *Xa3* and *Xa21* were amplified in both JJ623 and HR27195. M; DNA ladder, J; JJ623, H; HR27195.

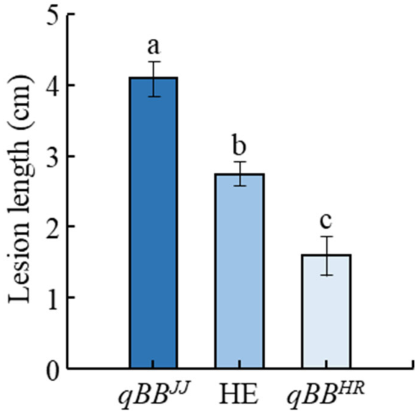

Figure S2. Frequency of *qBBR11* allele type in JJ623/HR27195 F_2 population. In the F_2 population, $qBBR11^{JJ623}qBBR11^{JJ623}$, $qBBR11^{JJ623}qBBR11^{HR27195}$ (HE), and $qBBR11^{HR27195}qBBR11^{HR27195}$ were separated 24:40:26, respectively. *qBBR11* in the F_2 population was suitable for the theoretical separation ratio of 1:2:1 (X^2 = 1.20, *P*-value = 0.549). And the lesion lengths of $qBBR11^{JJ623}qBBR11^{JJ623}$, $qBBR11^{JJ623}qBBR11^{HR27195}$, $qBBR11^{HR27195}qBBR11^{HR27195}$ were 4.1 cm, 2.8 cm, and 1.6 cm, respectively. The allele type of $qBBR11^{JJ623}$ regulate as an increase in lesion length.
